# Dysregulation of the miR-30a/BiP axis by cigarette smoking accelerates oral cancer progression

**DOI:** 10.1186/s12935-021-02276-1

**Published:** 2021-10-30

**Authors:** Chu-Yen Chien, Ying-Chen Chen, Chien‑Hsing Lee, Jia-Rong Wu, Tsai-Wang Huang, Ren-Yeong Huang, Wan-Chien Cheng, Alexander Cheng-Ting Hsieh, Yi-Shing Shieh

**Affiliations:** 1 Graduate Institute of Medical Sciences, National Defense Medical Center, Taipei City, Taiwan; 2 Molecular and Cell Biology, Taiwan International Graduate Program, Academia Sinica and Graduate Institute of Life Science, National Defense Medical Center, Taipei City, Taiwan; 3 Division of Endocrinology and Metabolism, Department of Internal Medicine, Tri-Service General Hospital, National Defense Medical Center, Taipei City, Taiwan; 4 Department and Graduate Institute of Biochemistry, National Defense Medical Center, Taipei City, Taiwan; 5 Graduate Institute of Life Sciences, National Defense Medical Center, Taipei City, Taiwan; 6 Division of Thoracic Surgery, Department of Surgery, Tri-Service General Hospital, National Defense Medical Center, Taipei City, Taiwan; 7 School of Dentistry, National Defense Medical Center, Taipei City, 114 Taiwan; 8 Department of Dentistry, Tri-Service General Hospital, National Defense Medical Center, Taipei City, Taiwan; 9 School of Traditional Chinese Medicine, Chang Gung University, Taoyuan City, Taiwan

**Keywords:** Chaperons, Cigarette smoking, miRNA, Oral squamous cell carcinoma, Vascular endothelial growth factor

## Abstract

**Background:**

Cigarette smoking is the most significant cause of oral cancer progression. Cigarette smoke condensate (CSC) has been shown to induce endoplasmic reticulum (ER) stress. Binding immunoglobulin protein (BiP) being as an ER stress regulator, has been reported to be implicated in malignant behaviors. Therefore, the aim of this study was to investigate the role of the ER stress-responsive protein, BiP, in CSC-induced oral squamous cell carcinoma (OSCC) malignancy.

**Methods:**

The biological role of BiP in CSC-induced tumor progression was investigated in OSCC cells (YD38 and SCC25) and in a tumor xenograft mouse model. The expressions of related genes were investigated using quantitative RT-PCR and Western blot analysis. Cell migration and invasion were assessed using scratch wound healing and Transwell invasion assays. The effects of conditioned media from OSCC cells on the angiogenic activities of endothelial cells were analyzed using a tube formation assay. The interaction between miR-30a and BiP mRNA was detected using a luciferase reporter assay.

**Results:**

Our results demonstrated that CSC increased the expression of BiP in time- and dose-dependent manners in YD38 and SCC25 cells, and that silencing BiP abrogated CSC-induced cell invasion and tumor-associated angiogenesis. Notably, the putative miR-30a binding site was observed in the 3′untranslated region (UTR) of BiP mRNA, and miR-30a suppressed BiP expression by targeting 3′UTR of BiP transcript. In addition, CSC increased the expression of BiP in OSCC cells by downregulating miR-30a. We also showed that BiP promoted invasion and tumor-associated angiogenesis by increasing the production and secretion of vascular endothelial growth factor in CSC-exposed OSCC cells. Moreover, BiP inhibition suppressed OSCC growth and reduced tumor vessel density in tumor-bearing mice administered with CSC.

**Conclusions:**

These observations suggest that epigenetic regulation of BiP via miR-30a downregulation is involved in CSC-induced OSCC progression.

## Background

Oral cancer is the sixth most prevalent cancer worldwide [1]. The main treatments for oral cancer are surgery, radiotherapy, and chemotherapy [2]. The current standard chemotherapeutic agents are cisplatin, 5-fluorouracil (5-FU), and doxorubicin [3]. According to its pathological features, oral cancer has been divided into different subtypes, namely oral squamous cell carcinoma (OSCC), melanoma (from melanocytes), mucoepidermoid carcinoma (from salivary glands), adenoid cystic carcinoma (from salivary glands), sarcomas (from palate), and lymphoma (from lymphocytes) [4]. The five-year survival rate of melanoma, mucoepidermoid carcinoma, adenoid cystic carcinoma, sarcomas, and lymphoma is 15, 50, 70, 45 and 50%, respectively [4–8]. Among oral cancer, OSCC accounts for over 90% of all cases [1, 9]. In spite of therapeutic advances in oral cancer, the five-year survival rate of OSCC patients has not remarkably improved, and is still around 50% [9]. A late diagnosis and distant metastasis remain clinical challenges for oral cancer patients [2]. Hence, it is important to understand the pathogenesis and disease progression of OSCC. Many risk factors have been linked to the development of oral cancer, including cigarette smoking, alcohol consumption, betel nut chewing, and viral infections [10]. Among them, cigarette smoking is the most common risk factor for oral malignancy [11]. Cigarette smoke is a complex mixture of over 7000 different compounds [12]. More than 70 of these compounds have been identified as carcinogens, which are highly associated with cancer development, such as oral and lung cancer [12, 13]. Smokers have been demonstrated to have a 5- to 25-fold higher risk of developing oral cancer compared with non-smokers [13]. Moreover, cigarette smoking has been shown to contribute to the cancer-associated transformation of oral epithelial cells [14]. In addition, cigarette smoke condensate (CSC) treatment has been reported to increase the invasion and migration of OSCC cells [15]. These findings suggest the role of cigarette smoking in the carcinogenesis and disease progression of oral cancer. Several oncogenic signaling pathways have been proposed to be involved in CSC-induced tumor malignancy, including PI3K/Akt, NFκB, Wnt/β-catenin and MAPK pathways [14, 16, 17]. In addition to the activation of oncogenic signaling, epigenetic dysregulation has been demonstrated to be a potential cause of cancer phenotypes, including head and neck, colorectal, lung, breast, and liver cancer [18, 19]. However, little is known about the role of epigenetic alterations in CSC-induced oral cancer progression.

Epigenetic modifications of chromatin through DNA methylation, histone modification, chromatin remodeling, nucleosome changes and non-coding RNA regulation are crucial regulators of gene expression [20]. Dysregulation of epigenetic regulation is frequently associated with cancer development and progression due to increased expressions of oncogenes and silencing of tumor suppressor genes [21]. Therefore, epigenetic therapeutic approaches have evolved as a novel strategy in cancer treatment [22]. A growing body of evidence has demonstrated that microRNAs (miRNAs) are aberrantly expressed in various types of cancer, such as acute myeloid leukemia (AML), oral, breast, lung, and colorectal cancer, and that they are critical in tumorigenesis and tumor metastasis [23–25]. miRNAs are a class of small non-coding RNA with a length ranging from 18 to 24 nucleotides, and they usually function as negative regulators of gene expression [26]]. Mechanistically, miRNAs regulate the post-transcriptional expression of genes by binding to the 3′untranslated region (UTR) of target mRNAs, leading to mRNA degradation or translation repression [26]. miRNA-30a (miR-30a) belongs to the miR-30 family, and it has been reported to suppress tumor malignant behaviors [27]. In OSCC, miR-30a has been shown to dampen cell proliferation, invasion, migration and cisplatin resistance both in vitro and in vivo [28, 29]. Clinically, the expression of miR-30a has been demonstrated to be lower in oral cancer tissues compared to adjacent non-cancerous tissues [2]. Of note, exposure to cigarette smoke has been reported to result in the downregulation of miR-30a in lung cancer [30, 31]. However, the underlying mechanisms of miR-30a in CSC-induced tumor malignancy, especially in oral cancer, have yet to be clearly defined.

Binding immunoglobulin protein (BiP) belongs to the HSP70 family. It is the master regulator of unfolded protein response during endoplasmic reticulum (ER) stress, and is crucial for protein folding and maturation in ER [32]. BiP has been demonstrated to be normally expressed at low basal levels in adult organs but at higher expressions in patients with cancer, such as pancreatic, breast, prostate, lung, and liver cancer, and to be associated with tumor malignancy [32, 33]. In addition, the upregulation of BiP has been closely correlated with poor outcomes in OSCC patients [34]. Recently, miRNAs have emerged as important regulators of the expressions of ER stress-responsive proteins, including BiP [35]. Previous studies have reported that miR-30a mimics can lead to a lower expression of BiP [36–38], which implies the regulatory role of miR-30a in BiP expression. Furthermore, cigarette smoke has been shown to upregulate BiP expression in both non-malignant and malignant cells [39–41]. However, the involvement of miR-30a-regulated BiP expression in CSC-induced oral cancer progression has not been investigated.

The aim of this study was to investigate the role of BiP in CSC-induced tumor malignancy and evaluate the involvement of miR-30a in CSC-mediated BiP expression in OSCC. Our results demonstrated that the miR-30a-BiP-vascular endothelial growth factor (VEGF) regulatory axis controlled tumor malignancy in tobacco-related oral cancer.

## Methods

### Cell lines and cell culture

The human OSCC cell lines, YD38 and SCC25, were cultured in RPMI medium (31800-022, GIBCO, Eggenstein, Germany) containing 10% fetal bovine serum (FBS) (10437-028, GIBCO, Eggenstein, Germany) and 1% penicillin–streptomycin-amphotericin B (PSA) (03-033, Biological industries, Cromwell, CT, USA). YD38 cells were obtained from Dr. Yook (Namseoul University, Korea). SCC25 cells were kindly provided by Dr. Shine-Gwo Shiah (National Institute of Cancer Research, National Health Research Institutes, Miaoli, Taiwan). Human umbilical vein endothelial cells (HUVECs) were obtained from ScienCell (San Diego, CA, USA) and maintained in endothelial cell medium (ECM) (1001, ScienCell, San Diego, CA, USA) supplemented with 5% FBS, 1% endothelial cell growth supplement (ECGS) and 1% penicillin–streptomycin solution. All cell lines were confirmed to be mycoplasma-free.

### Drugs and antibodies

CSC (NC1560725, Murty Pharmaceuticals, Lexington, KY, USA) was prepared by smoking University of Kentucky's 3R4F Standard Research Cigarettes dissolved in DMSO. VEGF recombinant protein (100–20) was purchased from Peprotech, Rocky Hill, NJ, USA. The primary antibodies used in the study included BiP (BD610978), E-cadherin (BD610181) and ZO-1 (BD610966, BD Biosciences, San Jose, CA, USA), fibronectin (ab32419) and VEGF (ab214424, Abcam, Cambridge, MA, USA), vimentin (CST5741) and GAPDH (CST5174, Cell Signaling, Beverly, MA, USA). The horseradish peroxidase (HRP)-conjugated secondary antibodies were purchased from Jackson ImmunoResearch Laboratories Inc. (West Grove, PA, USA).

### RNA interference

Both siRNA and miRNA were designed and synthesized by Dharmacon (Lafayette, CO, USA). The sequence of siRNA used to target BiP was 5’-CCACCAAGAUGCUGACAUU-3’, and for the non-targeted control 5’-UAGCGACUAAACACAUCAA-3’. The sequence of miR-30a mimics was 5’-UGUAAACAUCCUCGACUGGAAG-3’, and the negative control was 5’-CUCUUUCUAGGAGGUUGUGAUU-3’. The siRNA and miRNA were transfected into cells using GenMute siRNA transfection reagent (SL100568, SignaGen Laboratories, Ijamsville, MD, USA) according to the manufacturer’s instructions. In brief, cells were seeded in 6-well culture plates and incubated overnight. The cells were transfected with 10 nM siRNA or miRNA mimics, and subsequently used for the following experiments.

### RNA extraction and quantitative reverse transcription polymerase chain reaction (qRT-PCR)

Total RNA was extracted using TRIzol reagent (15596018, Invitrogen, Carlsbad, CA, USA). Complementary DNA (cDNA) was synthesized from mRNA using a high-capacity cDNA synthesis kit (4368813, Applied Biosystems, Carlsbad, CA, USA), and from miRNA using a qSTAR miRNA qPCR detection system (HP100042, OriGene, Rockville, MD, USA). qRT-PCR was carried out on a Roche LightCycler 480 system (Roche, Basel, Switzerland) using SYBR Green I Master mix (BIO-98005, Bioline Inc., Boston, MA, USA). The primer sequences are listed in Table 1. The results were normalized to either GAPDH for mRNA expression or U6 for miRNA expression.

**Table 1:** The primer sequences used for quantitative RT-PCR

Gene	5'-3'	Sequences
BiP	Forward	TGACATTGAAGACTTCAAAGCT
	Reverse	CTGCTGTATCCTCTTCACCAGT
miR-30a	Forward	AACATCCTCGACTGGAAG
	Reverse	GAACATGTCTGCGTATCTC
VEGF	Forward	GCCTTGCCTTGCTGCTCTAC
	Reverse	TGATTCTGCCCTCCTCCTTCTG
GAPDH	Forward	CCACATCGCTCAGACACCAT
	Reverse	TGACCAGGCGCCCAATA

### Western blot analysis

Proteins were extracted with radioimmunoprecipitation assay (RIPA) lysis buffer containing protease and phosphatase inhibitors (78447, Biological industries, Cromwell, CT, USA) for 30 min, and the protein concentration was measured using a BCA assay kit (23227, Thermo Fisher, Pittsburgh, PA, USA) according to the manufacturer’s protocols. The proteins were electrophoresed with 8% sodium dodecyl sulfate polyacrylamide gel electrophoresis (SDS-PAGE) and transferred onto polyvinylidene difluoride (PVDF) membranes (Millipore, Bedford, MA, USA). After blocking with 5% non-fat milk in 0.1% TBS-T for 1 h at room temperature, the membranes were incubated with specific primary antibodies overnight at 4℃. The primary antibodies were diluted in 5% non-fat milk and the dilutions were as follows: anti-BiP (1:4000), anti-E-cadherin (1:4000), anti-ZO-1 (1:1000), anti-fibronectin (1:1000), anti-VEGF (1:1000), anti-vimentin (1:1000), and anti-GAPDH (1:5000). The membranes were subsequently washed with 0.1% TBS-T and incubated with HRP-conjugated secondary antibodies for an additional 1 h. All bands were detected and analyzed using an ECL reagent kit (T-Pro Biotechnologies, Taipei City, Taiwan) and a UVP bioimaging system (Analytik Jena, Jena, Germany).

### Invasion assay

The invasion assay was carried out using Matrigel‐coated Transwell inserts with an 8-μm pore size (351152, Corning Inc., Corning, NY, USA). Cells were seeded at 2.5 × 10^4^ into the upper chambers of the Transwell inserts. After incubation for 24 h, the inserts were fixed with methanol and stained with propidium iodide (PI) (P4170, Sigma-Aldrich, St. Louis, MO, USA). The invaded cells across the membranes were visualized and photographed using a fluorescence microscope equipped with a camera at a magnification of 200X. The average number of invaded cells were counted from five microscopic fields per chamber of three independent experiments using ImageJ software.

### Scratch wound healing assay

Cell migration was assessed using a scratch wound healing assay. Cells were seeded in 6-well culture plates and cultured as a monolayer to confluence in complete culture media. Subsequently, the cell monolayers were scratched using a sterile micropipette tip and washed with PBS to remove suspended cells. The distance of the scratch closure was observed and photographed under a microscope equipped with a camera at 0 and 12 h. The wound area was determined using ImageJ software.

### Luciferase reporter gene assay

The 3′UTR of BiP containing the putative miR-30a binding site was cloned into the pMirTarget 3′UTR assay vector (SC216266, OriGene, Rockville, MD, USA). YD38 and SCC25 cells were transfected with pBiP-3′UTR plasmid using Lipofectamine 2000 transfection reagent (11668019, Invitrogen, Carlsbad, CA, USA). The activity of luciferase was measured using a luciferase assay kit (16184, Thermo Fisher, Pittsburgh, PA, USA) according to the manufacturer’s instructions. The red fluorescence intensity was used to normalize firefly luciferase activity.

### Enzyme-linked immunosorbent assay

The amount of VEGF in conditioned media (CM) harvested from YD38 and SCC25 cells was assayed using a human VEGF ELISA kit (BMS-277, Invitrogen, Carlsbad, CA, USA) according to the manufacturer's instructions.

### Tube formation assay

A tube formation assay was performed using an in vitro angiogenesis kit (ECM625, Merck Millipore, Darmstadt, Germany) according to the manufacture’s protocols. Briefly, HUVECs were seeded at 2 × 10^4^ in 96-well culture plates pre-coated with supplied ECMatrix, and incubated with CM harvested from OSCC cells. After incubation for 6 h, tube formation of HUVECs was observed using an inverted light microscope, and quantified by measuring the length of tube-like structures using an angiogenesis analyzer for ImageJ software.

### Histological and immunohistochemical staining

Tumor tissue sections were deparaffinized in xylene and rehydrated in a series of graded ethanol. Histological analysis of tumor tissues was carried out using hematoxylin and eosin (H&E) staining (HAE-1, ScyTek Laboratories, Logan, Utah, USA). Immunohistochemical staining was performed using a Novolink polymer detection system (RE7150-K, Leica Biosystems Newcastle Ltd., Newcastle, UK) according to the manufacturer's instructions. Heat-induced antigen retrieval was performed in Tris–EDTA buffer (pH 9.0) containing 10 mM Tris-base, 1 mM EDTA solution and 0.05% Tween-20 for 30 min. Endogenous peroxidase was quenched with peroxidase block, and non-specific protein binding was blocked using protein block. Subsequently, the slides were incubated with the appropriate primary antibodies overnight at 4 ℃, followed by incubation with the provided Novolink polymer for 1 h. The primary antibodies were diluted in protein block and the dilutions were as follows: anti-BiP (1:100), anti-VEGF (1:100), and anti-CD31 (1:100). The signals were developed with 3,3′-Diaminobenzidine (DAB) solution, and the nuclei were counterstained with hematoxylin. Quantification of histochemical staining was performed using ImageJ software.

### Animal study

Four-week-old male athymic nude mice were purchased from the National Laboratory Animal Center (Taipei, Taiwan), and housed in the animal center at the National Defense Medical Center (Taipei, Taiwan) with free access to food and water. All experiments were conducted in compliance with institutional guidelines approved by the Institutional Animal Care and Use Committee of the National Defense Medical Center. SCC25 (1.5 × 10^6^) cells transfected with scramble shRNA control (SCC25-shV) or BiP shRNA (SCC25-shBiP) (Academia Sinica RNAi core, Taipei, Taiwan) in sterile PBS mixed 1:1 with Matrigel (354234, Corning Inc., Corning, NY, USA) were subcutaneously injected into the right flank of the nude mice. When the tumors reached a volume of 100 mm^3^, the mice were randomly divided into vehicle control and treatment groups (n = 5 per group). CSC (20 mg/kg body weight) or PBS (vehicle control) was administered intraperitoneally every other day for 3 weeks as previously described [42, 43]. Tumor growth was monitored once a week using Vernier calipers, and the tumor volume was calculated according to the formula: V = (width^2^ × length)/2. The body weight of the nude mice was also recorded once a week. At the end of treatment, the mice were sacrificed using carbon dioxide (CO_2_) inhalation with a 30–70% flow rate in a chamber at least for 7 min. The tumors were dissected and weighed.

### Statistical analysis

The Student’s *t* test and one-way ANOVA followed by Bonferroni’s post hoc test were used to determine the statistical significance between two groups and among multiple groups, respectively. All statistical analyses and graphic representations were conducted using GraphPad Prism software (GraphPad Software Inc., San Diego, CA, USA). The results are expressed as the mean ± SEM. A P value < 0.05 was considered to indicate a significant difference.

## Results

### CSC induced the expression of BiP in OSCC cells in time- and dose-dependent manners

**Fig. 1:**
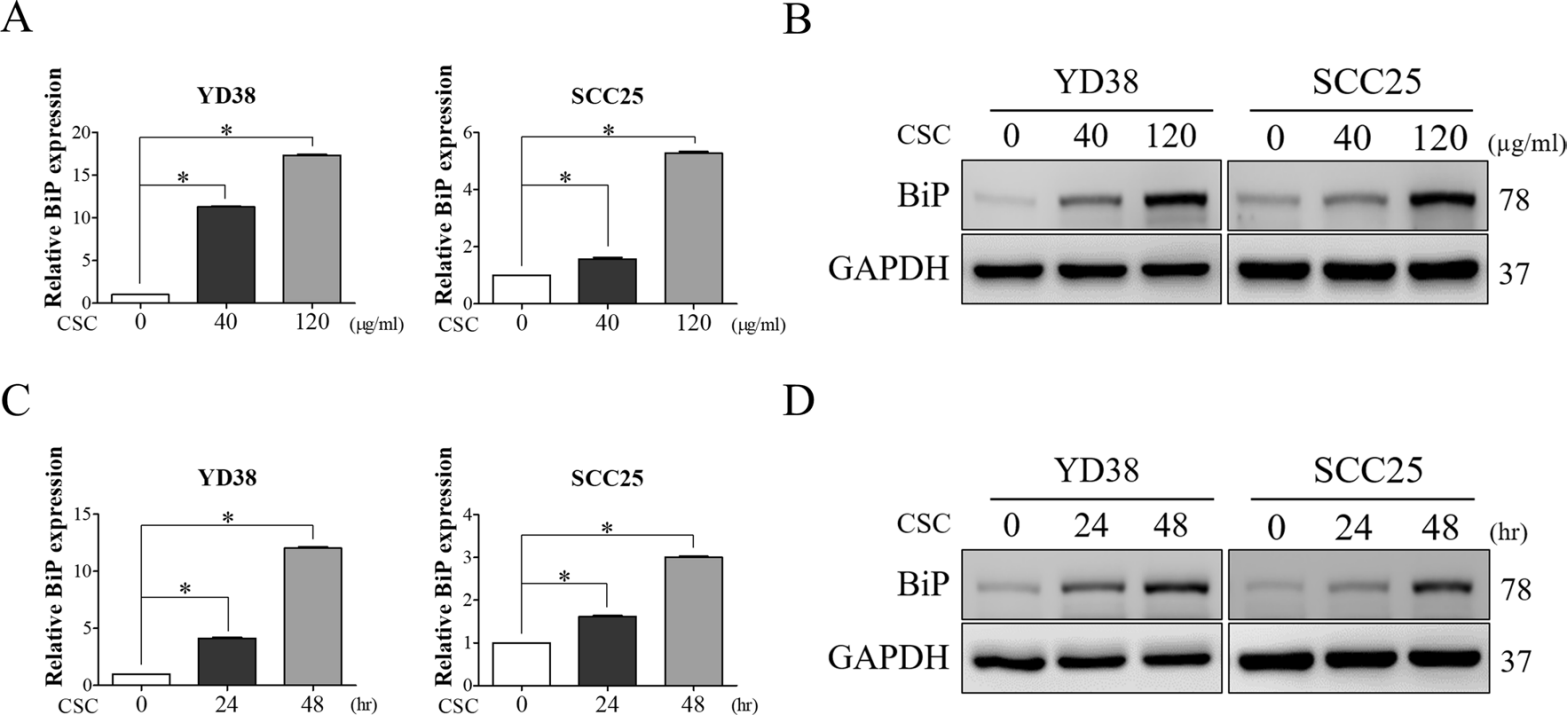
CSC significantly increased BiP expression in OSCC cells in dose- and time-dependent manners. **A** and **B** Dose-dependent effects (40 and 120 μg/ml) of CSC on the expression of BiP in YD38 and SCC25 cells were investigated by qRT-PCR and Western blot analysis. **C** and **D** Time-dependent effects of CSC (24 and 48 h) on the expression of BiP were examined by qRT-PCR and Western blot analysis. GAPDH was used as the internal control. All data are presented as the mean ± SEM. SEM, error bars. *P < 0.05 by one-way ANOVA followed by Bonferroni’s post hoc test

Cigarette smoking is a significant risk factor for oral cancer development and progression, and it has been demonstrated to trigger ER stress-associated responses [11, 44]. Therefore, the effect of CSC on the expression of BiP was investigated in OSCC cells (YD38 and SCC25) after exposure to various doses (40 and 120 μg/ml) of CSC for 48 h or 120 μg/ml CSC for 24 and 48 h. Dose- and time-dependent increases in the mRNA and protein expressions of BiP were observed in OSCC cells treated with CSC (Fig. 1). These results suggested the stimulatory effect of CSC on the expression of BiP in OSCC cells. Given that the most significant change in BiP expression was observed in cells exposed to 120 μg/ml of CSC for 48 h, this dose and time point of CSC treatment were subsequently used in the following experiments.

### CSC stimulated EMT change, migration, invasion and tumor-associated angiogenesis in OSCC cells

**Fig. 2:**
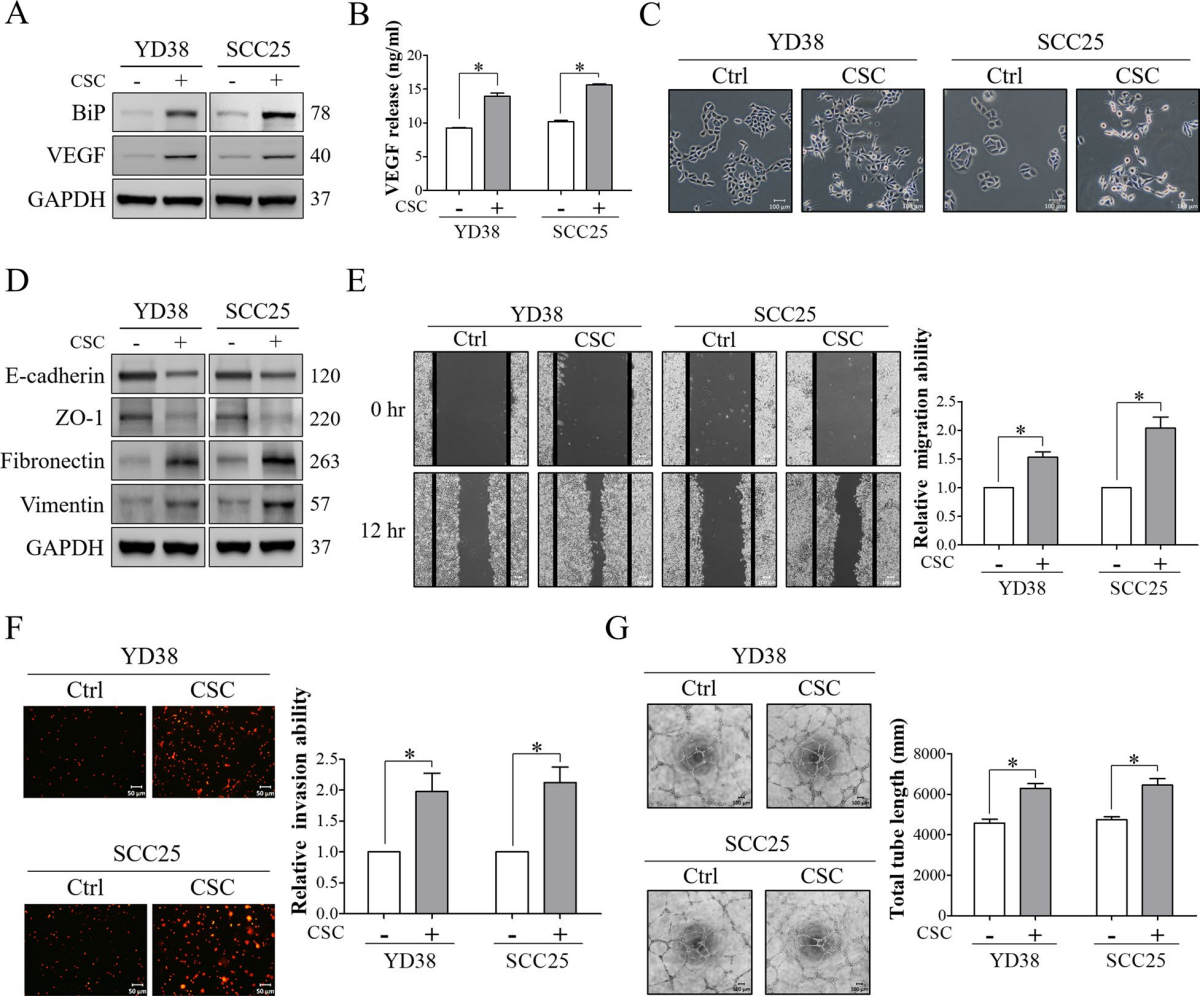
CSC promoted EMT, migration and invasion in OCSS cells, and increased tumor-associated tube formation of HUVECs. OSCC cells were treated with/without 120 μg/ml CSC for 48 h. **A** The expressions of BiP and vascular endothelial growth factor (VEGF) were analyzed by Western blot analysis. **B** The amount of VEGF in conditioned media (CM) derived from OSCC was examined by ELISA. **C** Representative images of epithelial–mesenchymal transition (EMT) morphological changes in OSCC cells after the indicated treatments (Scale bar, 100 μm). **D** The expressions of epithelial markers (E‐cadherin and ZO-1) and mesenchymal markers (fibronectin and vimentin) were detected by Western blot analysis. **E** The migratory ability of OSCC cells was evaluated using a wound-healing assay. Representative images were taken at 0 and 12 h after wound scratching (Scale bar, 100 μm). Solid black lines indicate wound borders. Quantification of wound closure was determined using ImageJ software. The ability of migration was calculated as the average reduction in area at 12 h compared to 0 h. **F** The invasion ability was determined using a Transwell invasion assay. OSCC cells were seeded into the upper chamber of Matrigel-coated inserts. After 24 h, the invaded cells were fixed with methanol and stained with propidium iodide (PI). Representative images are shown (Scale bar, 50 μm). Quantification of the invaded cells was performed using ImageJ software. **G** Tube formation activities of HUVECs were evaluated using a tube formation assay. HUVECs were seeded in 96-well culture plates pre-coated with ECMatrix and incubated with CM harvested from OSCC cells with/without CSC treatment. After 6 h, tube formation was observed under an inverted microscope and photographed. Representative images are shown (Scale bar, 100 μm). Quantitative analysis of total tube length was conducted using an angiogenesis analyzer for ImageJ software. GAPDH was used as the internal control. All data are presented as the mean ± SEM. SEM, error bar. *P < 0.05 by Student’s *t* test

Previous studies have reported that BiP is involved in the regulation of tumor function, including epithelial–mesenchymal transition (EMT) change, migration, invasion, and tumor-associated angiogenesis, and that VEGF is a downstream regulator of BiP participating in these pathological processes [45–47]. Therefore, the effect of CSC on the expression of VEGF and malignant behaviors were evaluated in YD38 and SCC25 cells after treatment with 120 μg/ml CSC for 48 h. We found that CSC increased the expression of VEGF in OSCC cells, and that the levels of secreted VEGF were significantly higher in the CM collected from OSCC cells incubated with CSC compared to those from the control cells (Fig. 2A, B). OSCC cells exposed to CSC displayed an elongated, spindle-shaped morphology and loose cell–cell adhesion compared to the control cells (Fig. 2C). To further examine whether these morphological changes were consistent with the molecular characteristics of EMT, the expressions of epithelial (E-cadherin and ZO-1) and mesenchymal (fibronectin and vimentin) markers were analyzed in OSCC cells after exposure to CSC. As shown in Fig. 2D, CSC promoted EMT in OSCC cells as evidenced by a loss of epithelial markers and induction of mesenchymal markers. Migration and invasion were also increased in OSCC cells after treatment with CSC (Fig. 2E, F). Furthermore, CM harvested from CSC-exposed cells significantly stimulated tube formation of endothelial cells (HUVECs) (Fig. 2G). These results indicated that malignant behaviors, including EMT, migration, invasion and angiogenesis, were significantly stimulated by treating OSCC cells with the indicated dose and time point of CSC.

### BiP inhibition suppressed CSC-induced OSCC invasion and tumor-associated angiogenesis by downregulating VEGF

**Fig. 3:**
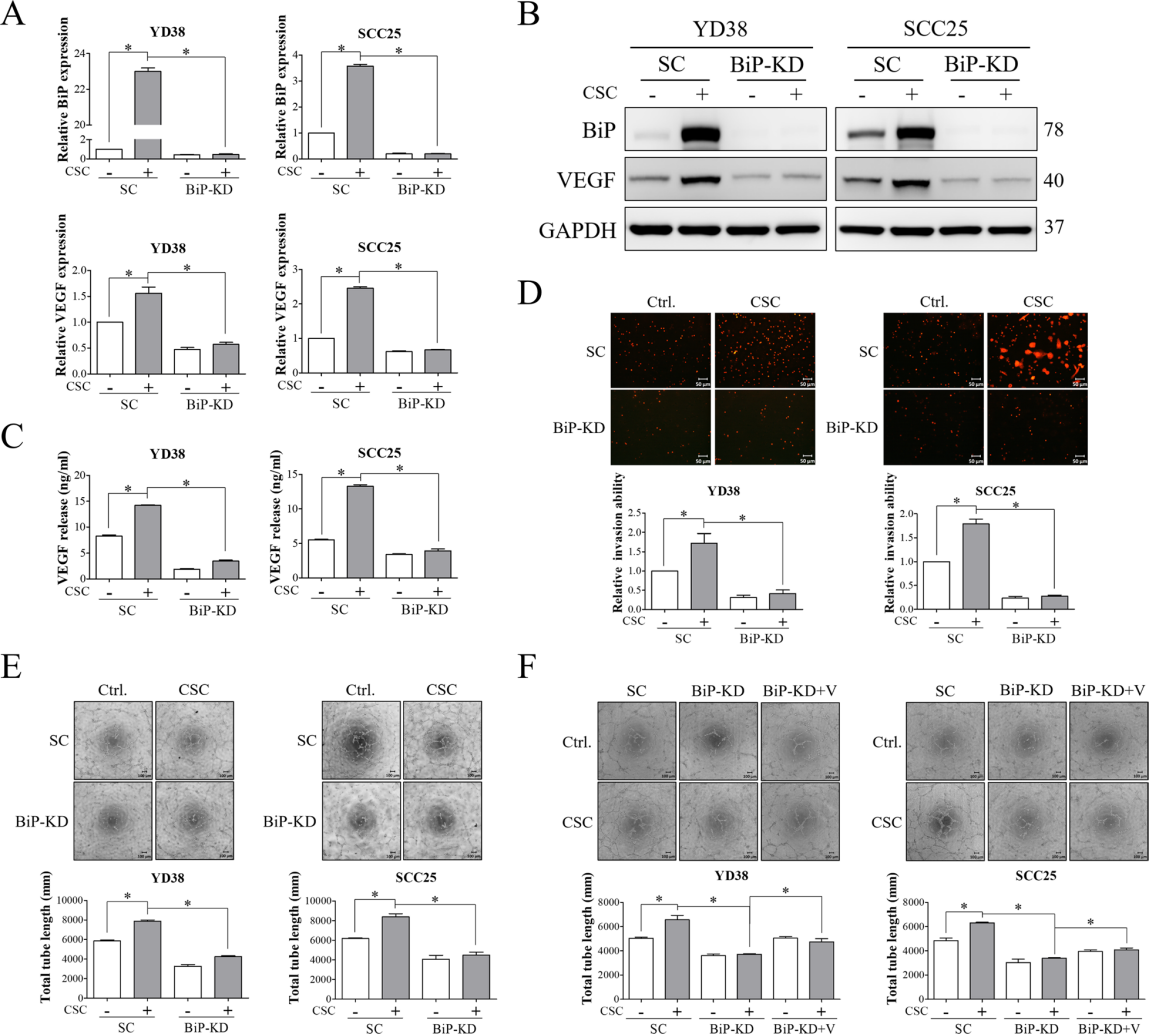
BiP modulated CSC-induced invasion of OSCC cells and tumor-associated angiogenesis by upregulating VEGF. YD38 and SCC25 cells transfected with either non-targeting siRNA or BiP siRNA were treated with 120 μg/ml CSC for 48 h. **A** and **B** The mRNA and protein expressions of BiP and VEGF were detected by qRT-PCR and Western blot analysis, respectively. **C** ELISA was used to detect the amount of VEGF in conditioned media (CM) harvested from OSCC cells. **D** The ability of cell invasion was examined using a Transwell invasion assay. OSCC cells after the indicated treatments were seeded into the inserts pre-coated with Matrigel and incubated for 24 h. Subsequently, the cells were fixed with methanol and stained with propidium iodide (PI). Representative images are shown (Scale bar, 50 μm). The PI-stained cells were quantified using ImageJ software. **E** The effect of OSCC cell-derived CM on the angiogenicity of HUVECs was examined using a tube formation assay. HUVECs in CM from OSCC cells were seeded in 96-well culture plates containing ECMatrix. Tube formation of HUVECs was observed and imaged after 6 h (Scale bar, 100 μm). Total tube length was quantified using an angiogenesis analyzer for ImageJ software. **F** The downstream role of VEGF in BiP-mediated tumor-associated tube formation of HUVECs was evaluated by addition of 10 ng/ml of VEGF recombinant protein into CM harvested from BiP-silenced cells after treated with CSC. HUVECs incubated with OSCC-derived CM with/without VEGF addition were seeded in 96-well culture plates containing ECMatrix. After 6 h, tube formation of HUVECs was observed and photographed under a microscopy equipped with a camera (Scale bar, 100 μm). Total tube length was determined using an angiogenesis analyzer for ImageJ software. SC, non-targeting siRNA-transfected cells. BiP-KD, BiP siRNA-transfected cells. GAPDH was used as the internal control. V, VEGF recombinant protein. All data are presented as the mean ± SEM. SEM, error bar. *P < 0.05 by one-way ANOVA followed by Bonferroni’s post hoc test

To further investigate the role of BiP in CSC-promoted tumor function, YD38 and SCC25 cells with/without BiP silencing were subjected to CSC treatment. As shown in Fig. 3A and B, CSC significantly increased the expressions of BiP and VEGF, and these effects were suppressed in BiP-silenced cells. In addition, depletion of BiP expression resulted in a decrease in the amount of VEGF in CM harvested from CSC-treated OSCC cells (Fig. 3C). CSC-induced cell invasion and tumor-associated tube formation of HUVECs were also inhibited by knockdown of BiP expression (Fig. 3D and E). Moreover, addition of VEGF into the CM derived from BiP-silenced cells after CSC treatment markedly rescued the CSC-promoted tumor-associated angiogenesis (Fig. 3F). These results indicated that BiP modulated CSC-stimulated malignant behaviors by increasing the production and secretion of VEGF.

### miR-30a downregulation epigenetically regulated BiP expression and malignant behaviors in CSC-exposed OSCC cells

**Fig. 4:**
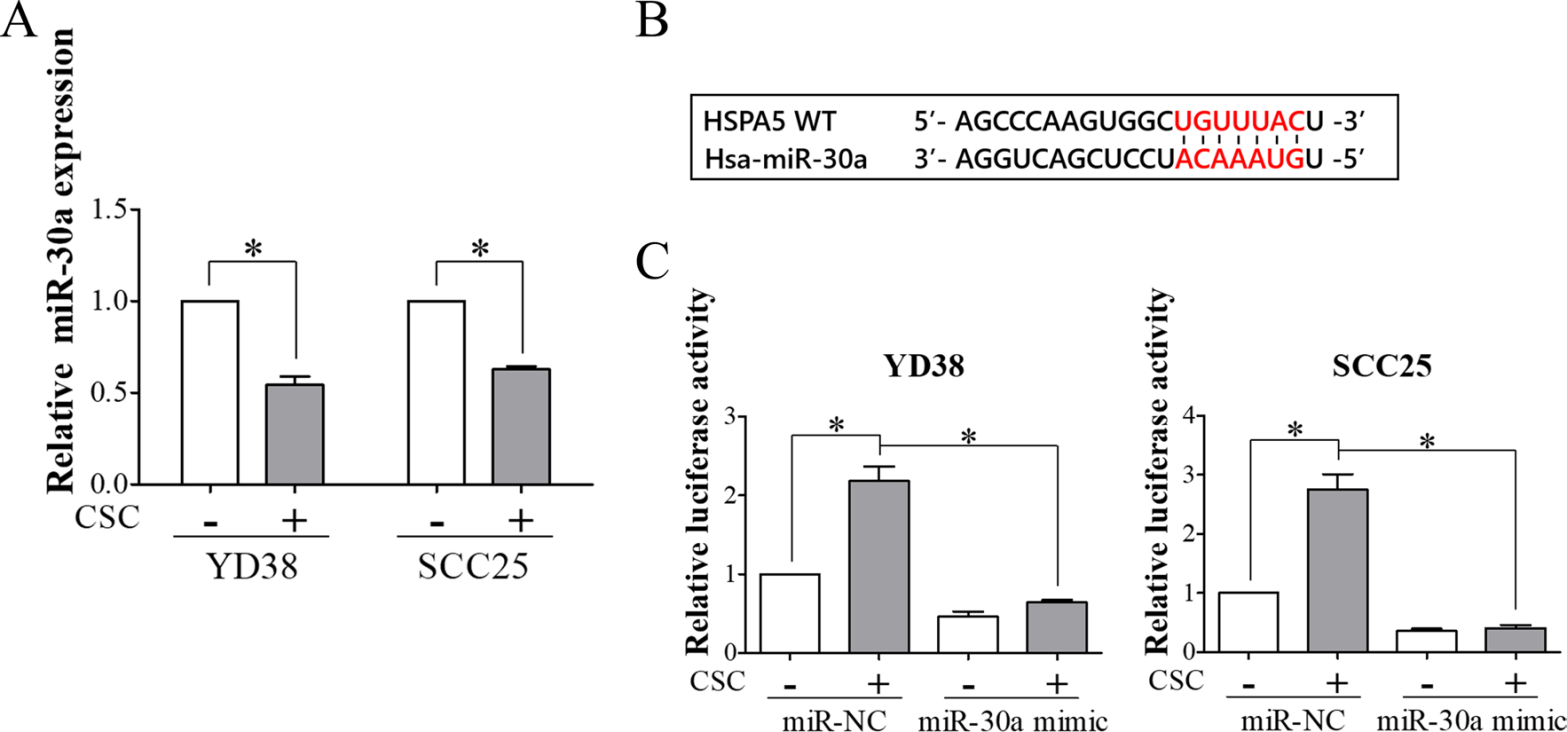
CSC induced BiP expression in OSCC cells by downregulating miR-30a. **A** YD38 and SCC25 cells were treated with either vehicle control or 120 μg/ml CSC for 48 h. The expression of miR-30a was evaluated using qRT-PCR. **B** Sequence alignment of miR-30a with the target sequence on the 3-UTR of BiP transcript. **C** The direct target relationship between miR-30a and BiP was confirmed using a luciferase assay. OSCC cells transfected with negative control (miR-NC) or miR-30a mimics were further transfected with pBiP-3'UTR plasmids followed by CSC treatment. Luciferase activity was detected using a luciferase assay. All data are presented as the mean ± SEM. SEM, error bar. *P < 0.05 by Student’s *t* test or one-way ANOVA followed by Bonferroni’s post hoc test

**Fig. 5:**
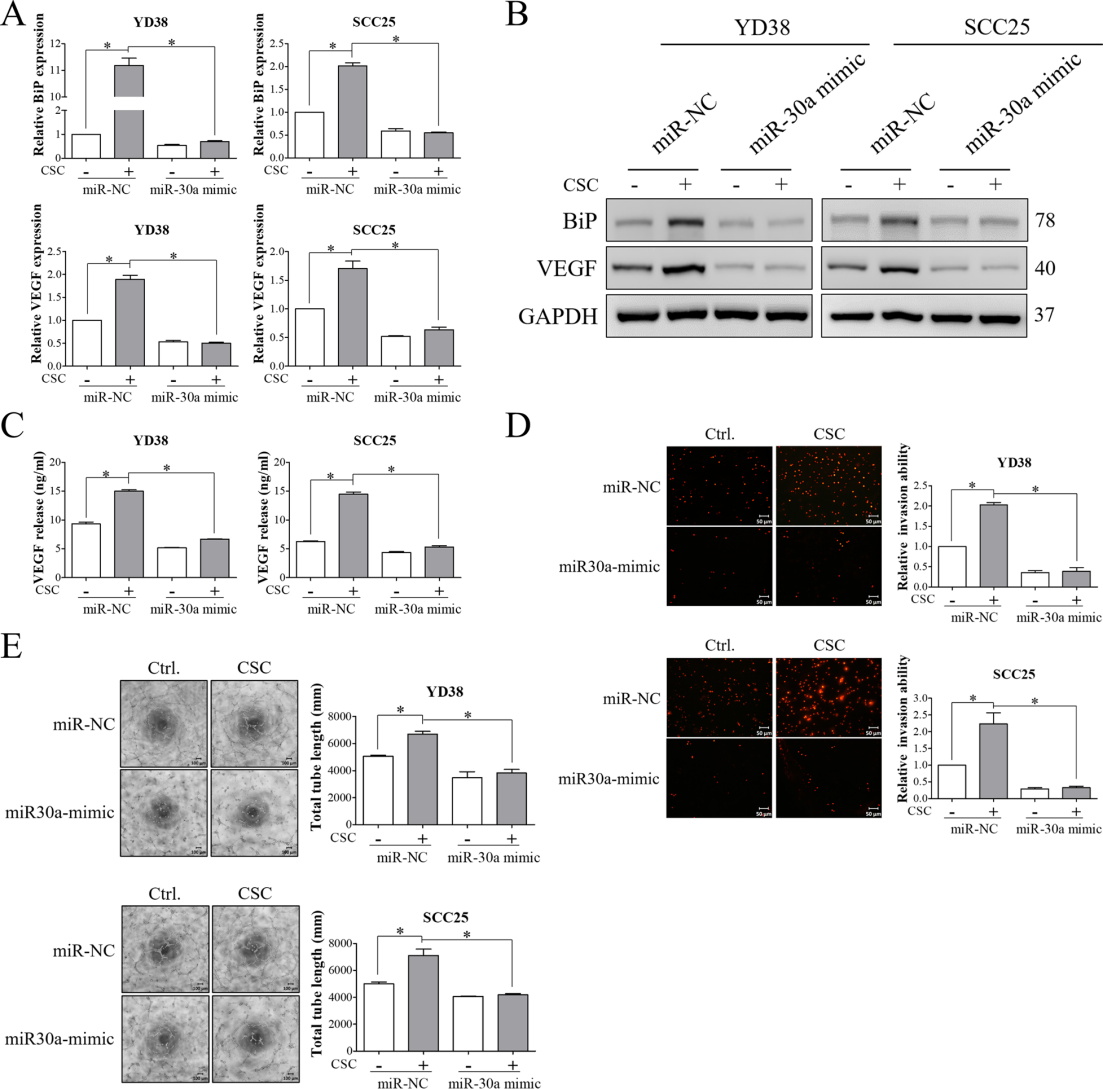
miR-30a was involved in CSC-induced BiP expression and OSCC malignancy. YD38 and SCC25 cells transfected with miR negative control (miR-NC) or miR-30a mimics were treated with 120 μg/ml CSC for 48 h. **A** and **B** The expressions of BiP and VEGF were examined by qRT-PCR and Western blot analysis. **C** The secretion of VEGF from OSCC cells was detected by ELISA. **D** The invasion ability was evaluated using a Transwell invasion assay. OSCC cells after the indicated treatments were allowed to invade through Matrigel-coated inserts for 24 h. The invaded cells were then fixed with methanol and stained with propidium iodide (PI). Representative images are shown (Scale bar, 50 μm). The amount of PI-stained cells was determined using ImageJ software. **E** The effect of OSCC cell-derived conditioned media (CM) on the angiogenic activities of HUVECs was determined using a tube formation assay. Representative images show the tube formation of HUVECs in CM from OSCC cells at 6 h (Scale bar, 100 μm). Total tube length was analyzed using an angiogenesis analyzer for ImageJ software. GAPDH was used as the internal control. All data are presented as the mean ± SEM. SEM, error bar. *P < 0.05 by one-way ANOVA followed by Bonferroni’s post hoc test

miR-30a has been demonstrated to suppress the expression of BiP in renal cell carcinoma [36]. To investigate the involvement of miR-30a in CSC-induced BiP expression, the effect of CSC on the expression of miR-30a was evaluated. As demonstrated in Fig. 4A, the expression of miR-30a was downregulated in YD38 and SCC25 cells after treatment with CSC. Notably, we observed one putative binding site of miR-30a located in the 3′UTR of BiP mRNA (Fig. 4B). To analyze the effect of CSC on the interaction between miR-30a and BiP mRNA, luciferase activity was detected in miR-30a-overexpressing cells transfected with pBiP-3′UTR reporter plasmids after CSC exposure. Increased luciferase activity was observed in OSCC cells after CSC exposure, and this effect was reversed by miR-30a overexpression (Fig. 4C). Moreover, the stimulatory effect of CSC on the expressions of BiP and VEGF was suppressed in miR-30a-overexpressing cells (Fig. 5A and B). Overexpression of miR-30a also decreased the secretion of VEGF from CSC-exposed cells (Fig. 5C). Transwell invasion and tube formation assays showed that CSC-induced invasion and angiogenesis were inhibited by overexpressing miR-30a (Fig. 5D and E). These results indicated the regulatory role of miR-30a in CSC-induced BiP expression and tumor malignancy in OSCC.

### BiP inhibition reversed CSC-induced OSCC growth and angiogenesis in tumor-bearing mice

**Fig. 6:**
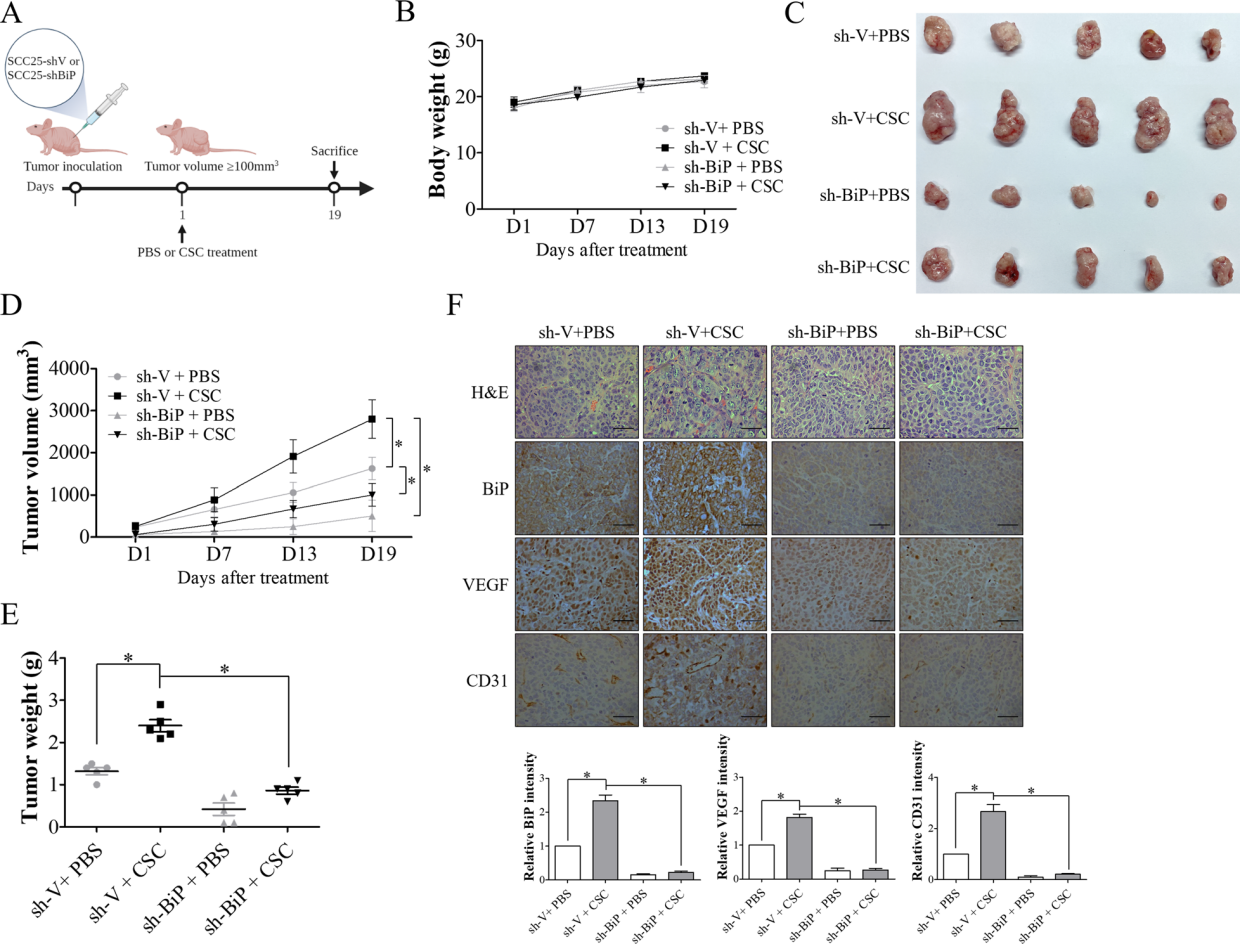
BiP inhibition suppressed CSC-stimulated OSCC progression in nude mice. SCC25 cells transfected with scramble shRNA control (SCC25-shV) or BiP shRNA (SCC25-shBiP) (1.5 × 10^6^ cells/mice) were injected subcutaneously into nude mice. Treatment was initiated when the tumor volume reached approximately 100 mm^3^, and the tumor-bearing mice were given intraperitoneal injections of either PBS or 20 mg/kg CSC every other day for 3 weeks. Body weight and tumor volumes were measured once every week. Following treatment, the mice were sacrificed and the tumor tissues were subjected to immunohistochemical staining for BiP, VEGF and CD31. CD31 was served as a marker of blood vessels. **A** Timeline of the animal experiments. **B** Average body weight changes of the mice. **C** Images of excised tumors (n = 5 per group). **D** and **E** Average tumor growth curve and tumor weight of OSCC tumors in the mice with different treatments. **F** Representative images showing the hematoxylin and eosin (H&E) staining and the immunohistochemical staining of BiP, VEGF and CD31 in tumor tissues. The relative intensity of immunohistochemical staining was determined using ImageJ software. All data are presented as the mean ± SEM. SEM, error bar. *P < 0.05 by one-way ANOVA followed by Bonferroni’s post hoc test. Scale bar, 50 μm

In order to further clarify the role of BiP in CSC-induced OSCC progression, mice were subcutaneously implanted with SCC25-shV or SCC25-shBiP cells followed by treatment with either PBS or CSC (Fig. 6A). There were no significant differences in body weight changes among the mice in all groups (Fig. 6B). Tumor growth was markedly increased in the mice injected with CSC compared to those injected with PBS, and the stimulatory effect of CSC on cell growth was inhibited by BiP silencing (Fig. 6C–E). As shown in results of H&E staining, tumor tissues from mice treated with CSC were characterized with increased mitogenic and angiogenic properties, and that this effect was suppressed by BiP inhibition (Fig. 6F). Furthermore, immunohistochemical staining showed that the expressions of VEGF and CD31 were increased in the tumor tissues from mice treated with CSC. However, these effects were reversed by depletion of BiP expression (Fig. 6F). These in vivo results further confirmed that BiP was involved in CSC-induced tumor growth and angiogenesis in OSCC.

## Discussion

Cigarette smoking is a significant carcinogenic factor for OSCC progression. However, the effect of cigarette smoking on the molecular pathogenesis in OSCC is unclear. Our results demonstrated that CSC stimulated invasion and tumor-associated angiogenesis by inducing the expression of BiP, and a subsequent increase in VEGF expression. In addition, miR-30a participated in the epigenetic regulation of BiP expression after CSC exposure.

Previous studies have demonstrated that miR-30a can function as an oncogene or a tumor suppressor gene in cancer [27]. Liu et al. reported that miR-30a was downregulated in melanoma cells and tissues, and that the overexpression of this molecule significantly suppressed cell proliferation, invasion and migration in melanoma [48]. In colorectal cancer, miR-30a has been shown to inhibit EMT and cell motility, and to be inversely correlated with tumor stage and status of lymph node metastasis [49]. These findings are consistent with our results that miR-30a acts as a tumor suppressor in cancer. On the other hand, miR-30a activated by the Wnt/β-catenin pathway has been demonstrated to promote invasion of glioma cells [50]. Moreover, miR-30a has been reported to be upregulated in metastatic nasopharyngeal carcinoma (NPC) compared to primary NPC tumors [51]. The overexpression of miR-30a has also been shown to significantly increase the invasion and metastasis of NPC cells, and to be associated with a poor prognosis in NPC patients [51]. These findings indicate that the biological role of miR-30a may be different depending on the type of cancer. Hence, a better understanding of the downstream target genes regulated by miR-30a is needed to clarify its functional role in cancer. miR-30a has been demonstrated to have a suppressive effect on OSCC progression by targeting downstream oncogenes [2, 28]. For example, miR-30a has been shown to sensitize OSCC cells to cisplatin by modulating the expression of the anti-apoptotic molecule, Bcl-2 [28]. Ruan et al. reported that miR-30a was associated with decreased cell migration and invasion by suppressing the expression of fibroblast activation protein (FAP) in OSCC [2]. Moreover, miR-30a has been shown to inhibit the growth of OSCC cells by repressing the expression of DNMT3B [52]. Notably, our results demonstrated that BiP was the downstream target of miR-30a, and that a lower expression of BiP was observed in miR-30a-overexpressing cells after CSC exposure. Therefore, our findings provide evidence that miR-30a plays a tumor suppressive role in OSCC, and that downregulation of miR-30a and a consequent increase in the expression of BiP are involved in CSC-induced tumor progression.

Our results further demonstrated that BiP was involved in CSC-induced invasion and angiogenesis in OSCC. Moreover, we also found that BiP promoted VEGF production and secretion in OSCC cells after CSC exposure. Traditionally, BiP is regarded to be a molecular chaperone in ER controlling protein folding and regulating unfolded protein response [53]. Increasing evidence has demonstrated that BiP is a multifunctional protein located in different cellular compartments, and that it is implicated in tumor malignancy [54]. For example, Shu et al. reported that mitochondrial BiP associated with Raf-1 inhibited cytochrome c release from mitochondria, and that it protected cells from ER stress-induced apoptosis in non-small cell lung cancer [55]. In addition, Casas showed that relocation of BiP to the plasma membrane could act as a signal receptor and activate downstream oncogenic pathways, thereby increasing cell proliferation and conferring chemoresistance in prostate and gastric cancer [56, 57]. Moreover, BiP has been detected in CM from hepatocellular carcinoma (HCC) cells and serum samples of HCC patients [58]. Secreted BiP then physically interacts with EGFR, activates EGFR-SRC-STAT3 signaling, and contributes to resistance to targeted therapy [58]. Several factors have been reported to trigger cellular trafficking of BiP [59–62]. For example, inflammatory cytokines and the ER stress inducer, thapsigargin, have been shown to induce membrane translocation of BiP in pancreatic beta cells and HeLa cervical cancer cells, respectively [59, 60]. Moreover, the ectopic expression of BiP has been shown to lead to cell surface localization of this molecule independently of ER stress [61]. Of note, increased secretion of BiP into bronchoalveolar lavage fluid has been observed in cigarette smokers compared to non-smokers [62]. Therefore, further studies are warranted to evaluate the effect of CSC on the subcellular distribution of BiP and its biological role in OSCC.

## Conclusions

**Fig. 7:**
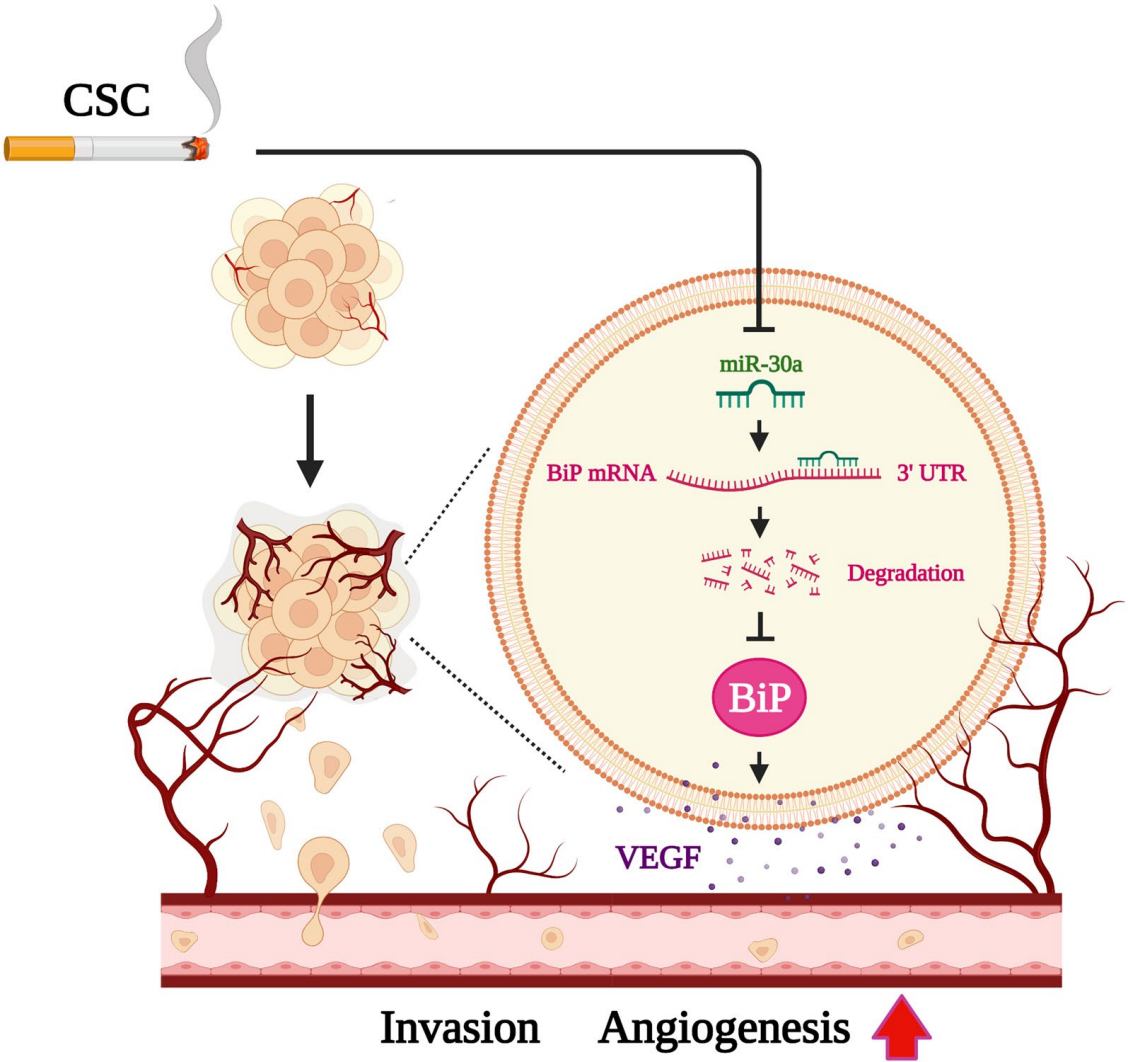
CSC stimulated BiP expression and malignant behaviors by downregulating miR-30a. CSC promoted cell invasion and tumor-associated angiogenesis by inducing the expression of BiP. Mechanistically, downregulation of miR-30a was involved in the epigenetic regulation of BiP expression in OSCC cells after exposure to CSC. In addition, BiP stimulated malignant behaviors of CSC-exposed OSCC cells through increased VEGF production and secretion

In conclusion, our results demonstrated that BiP was involved in CSC-induced malignant behaviors both in vitro and in vivo. Mechanistically, miR-30a regulated the expression of BiP in OSCC cells after CSC exposure. In addition, BiP increased the expression and secretion of VEGF in CSC-treated OSCC cells (Fig. 7). These findings not only provide a non-canonical mechanism for the regulation of the ER stress responsive protein, BiP, in OSCC cells after exposure to CSC, but also offer a potential therapeutic strategy for tobacco-related oral cancer.

## Data Availability

The datasets used and/or analysed during the current study are available from the corresponding author on reasonable request.

## Abbreviations

BiP: Binding immunoglobulin protein
CM: Conditioned media
CSC: Cigarette smoke condensate
DAB: Diaminobenzidine
ECGS: Endothelial cell growth supplement
ECM: Endothelial cell medium
ELISA: Enzyme-linked immunosorbent assay
EMT: Epithelial–mesenchymal transition
ER: Endoplasmic reticulum
FAP: Fibroblast activation protein
FBS: Fetal bovine serum
GAPDH: Glyceraldehyde 3-phosphate dehydrogenase
HCC: Hepatocellular carcinoma
HRP: Horseradish peroxidase
HUVECs: Human umbilical vein endothelial cells
miRNAs: MicroRNAs
NPC: Nasopharyngeal carcinoma
OSCC: Oral squamous cell carcinoma
PI: Propidium iodide
PSA: Penicillin–streptomycin-amphotericin B
PVDF: Polyvinylidene difluoride
qRT-PCR: Quantitative reverse transcription polymerase chain reaction
RIPA: Radioimmunoprecipitation assay
SDS-PAGE: Sodium dodecyl sulfate polyacrylamide gel electrophoresis
UTR: Untranslated region
VEGF: Vascular endothelial growth factor
